# N-glycosylation Profiling of Colorectal Cancer Cell Lines Reveals Association of Fucosylation with Differentiation and Caudal Type Homebox 1 (CDX1)/Villin mRNA Expression* [Fn FN2]

**DOI:** 10.1074/mcp.M115.051235

**Published:** 2016-01

**Authors:** Stephanie Holst, Anna J. M. Deuss, Gabi W. van Pelt, Sandra J. van Vliet, Juan J. Garcia-Vallejo, Carolien A. M. Koeleman, André M. Deelder, Wilma E. Mesker, Rob A. Tollenaar, Yoann Rombouts, Manfred Wuhrer

**Affiliations:** From the ‡Center for Proteomics and Metabolomics,; §Department of Surgery, and; ¶Department of RheumatologyLeiden University Medical Center, Leiden, The Netherlands;; ‖Department of Molecular Cell Biology and Immunology and; **Division of BioAnalytical Chemistry, VU University Medical Center, Amsterdam, The Netherlands;; ‡‡Univ. Lille, CNRS, UMR 8576, UGSF, Unité de Glycobiologie Structurale et Fonctionnelle, F 59 000 Lille, France

## Abstract

Various cancers such as colorectal cancer (CRC) are associated with alterations in protein glycosylation. CRC cell lines are frequently used to study these (glyco)biological changes and their mechanisms. However, differences between CRC cell lines with regard to their glycosylation have hitherto been largely neglected. Here, we comprehensively characterized the *N*-glycan profiles of 25 different CRC cell lines, derived from primary tumors and metastatic sites, in order to investigate their potential as glycobiological tumor model systems and to reveal glycans associated with cell line phenotypes. We applied an optimized, high-throughput membrane-based enzymatic glycan release for small sample amounts. Released glycans were derivatized to stabilize and differentiate between α2,3- and α2,6-linked *N*-acetylneuraminic acids, followed by *N*-glycosylation analysis by MALDI-TOF(/TOF)-MS. Our results showed pronounced differences between the *N*-glycosylation patterns of CRC cell lines. CRC cell line profiles differed from tissue-derived *N*-glycan profiles with regard to their high-mannose *N*-glycan content but showed a large overlap for complex type *N*-glycans, supporting their use as a glycobiological cancer model system. Importantly, we could show that the high-mannose *N*-glycans did not only occur as intracellular precursors but were also present at the cell surface. The obtained CRC cell line *N*-glycan features were not clearly correlated with mRNA expression levels of glycosyltransferases, demonstrating the usefulness of performing the structural analysis of glycans. Finally, correlation of CRC cell line glycosylation features with cancer cell markers and phenotypes revealed an association between highly fucosylated glycans and CDX1 and/or villin mRNA expression that both correlate with cell differentiation. Together, our findings provide new insights into CRC-associated glycan changes and setting the basis for more in-depth experiments on glycan function and regulation.

Colorectal cancer (CRC)^1^ is a very prevalent and heterogeneous pathology with highly variable disease progression and clinical outcome among patients. It is the third most common cancer in men and the second most common in women (1) with a highly stage-specific patient survival (2). Treatments are often curative for patients with local disease stages (stage I-II), whereas a 5-year survival of only 13% is observed in patients with distant metastasis (stage IV) (2). As CRC is often asymptomatic in the first years, unfortunately, only 40% of the patients are diagnosed at stage I-II, thus pointing to the urgent need of sensitive diagnostic tools for early detection and consequently effective, curative treatment (3). In this context, understanding the complex mechanisms of CRC is an overriding condition for the development of new, more efficient means of detection, treatment, and prognosis of the disease.

Altered glycosylation is a hallmark of cancer (4) and is known to occur with cancer progression (4, 5) as glycans are involved in many cancer-associated events such as adhesion, invasion, and cell signaling (6). As a result of altered glycan structures, cellular processes can be affected due to a change of interactions with glycan-binding proteins (7–9). Several CRC tissue-associated changes in *N*-glycans, *O*-glycans, and glycosphingolipid glycans have been reported and recently reviewed (7). For instance, *N*-glycans extracted from colorectal tumor tissues are characterized by an increase of sulfated glycans, (truncated) high-mannose-type glycans, and glycans containing sialylated Lewis type epitopes, while showing a decrease of bisection as compared with glycans from nontumor colorectal tissue of the same individuals (10). In accordance, elevated expression of sialyl Lewis A (NeuAcα2,3Galβ1,3[Fucα1,4]GlcNAc-R; NeuAc = *N*-acetylneuraminic acid, Gal = galactose, Fuc = fucose, GlcNAc = *N*-acetylglucosamine, R = rest) and pauci-mannosidic *N*-glycans (truncated high-mannose-type, Man1–4GlcNAc1–4GlcNAc; Man = mannose) was recently found to be correlated with poor prognosis in (advanced) colon carcinomas and *N*-glycomic profiling was successfully applied to distinguish colorectal adenomas from carcinomas (11).

Due to limitations in accessibility of tumor materials and possibilities of *in vivo* studies on a large scale, cancer cell lines represent a relevant alternative and are widely used as model systems for studying the molecular mechanisms associated with cancer outcome and progression. Since the early 1960s, colorectal cancer cell lines have been established with HT29, LoVo, LS-180, LS-174T, and Co115 representing the first continuous cell lines derived from colon tumors and xenografts (12–14). Major benefits of cancer cell lines are their continuous availability, their fast growth, and relatively easy handling, making them suitable also for high-throughput screenings (15) and a large range of experimental possibilities (16). Of note, advantages and limitations of cell lines have been recently reviewed (15).

In order to select suitable *in vitro* models, the characterization of molecular features and their comparison to tumor tissues are needed. A detailed Cancer Cell Line Encyclopedia was recently established containing a genomic dataset for 947 human cancer cell lines, from which 58 are colorectal cancer lineages (17). The Cancer Cell Line Encyclopedia includes data collections on genomic characterization, point mutation frequencies, DNA copy number, and mRNA expression levels. Comparison of these features between cell lines and primary tumors showed a high correlation in most cancer types, especially for colorectal cancer, suggesting that cell lines do represent tumor tissues quite reasonably at least on the genetic level. However, the number of publications characterizing cancer cell lines at a molecular level is far behind the number of articles using cancer cell lines as model systems (18), and only few studies have been conducted on whether *in vitro* cultured cell lines can serve as suitable models for human tumors (19–22). Furthermore, cell lines are well characterized genetically, but they are largely understudied with regard to their glycosylation profiles.

Here, we developed and optimized a new analytical method for the more sensitive and higher throughput *N*-glycome profiling of cells. This method is based on the release of *N*-glycans in a 96-well plate format from a PVDF-membrane (23) starting from a low number of cells (250,000 cells), the chemical derivatization of released *N*-glycans enabling the stabilization and discrimination of α2,3- and α2,6-linked *N*-acetylneuraminic acids (24), followed by registration of the *N*-glycans by MALDI-TOF(/TOF)-MS. The method was applied to characterize the *N*-glycome of 25 different colorectal cell lines in a fast and robust manner, including biological and technical replicates for all the cell lines. We obtained the comprehensive *N*-glycan profiles of 21 cell lines derived from primary tumors, two from lymph node metastases, one from a lung metastasis, and one from ascites fluid to assess their potential as glycobiological tumor model systems. Cancer cell line glycosylation features were then correlated with cancer cell markers and phenotypes as well as glycosyltransferase expressions. This study provides new insights into colon-cancer-associated glycan changes and sets a basis for studies into the functions of *N*-glycans in CRC with cell lines as model systems.

## MATERIALS AND METHODS

### 

#### 

##### Materials

Ammonium formiate, 2-aminobenzoic acid (AA), 2-picoline borane, dimethylsulfoxid (DMSO), 8 m guanidine hydrochloride (GuHCl), 1-hydroxybenzotriazole hydrate (HOBt), 50% sodium hydroxide, super DHB matrix (2-hydroxy-5-methoxy-benzoic acid and 2,5-dihydroxybenzoic acid, 1:9), and trifluoroacetic acid (TFA) were obtained from Sigma-Aldrich (St. Louis, MO). HPLC SupraGradient acetonitrile (ACN) was obtained from Biosolve (Valkenswaard, The Netherlands). Dithiothreitol (DTT), ethanol, sodium bicarbonate (NaHCO_3_), and glacial acetic acid were from Merck (Darmstadt, Germany) and 1-ethyl-3-(3-dimethylaminopropyl) carbodiimide (EDC) from Fluorochem (Hadfield, UK). Peptide *N*-glycosidase F (PNGase F) was purchased from Roche Diagnostics (Mannheim, Germany). Recombinant PNGase F from *Flavobacterium meningosepticum* was a kind gift from Rick R. Drake (Medical University of South Carolina, SC) and Anand S. Mehta (Drexel University College of Medicine, PA). It was expressed and purified as described previously (25) and is commercially available from Bulldog Bio (Portsmouth, NH) as PNGase F Prime™. The peptide calibration standard was purchased from Bruker Daltonics (Bremen, Germany). MultiScreen® HTS 96 multiwell plates (pore size 0.45 μm) with high protein-binding membrane (hydrophobic Immobilon-P PVDF membrane) were purchased from Millipore (Amsterdam, The Netherlands), conical 96-well Nunc plates from Thermo Scientific (Roskilde, Denmark). Hepes-buffered RPMI 1640 and Dulbecco's Modified Eagle (DMEM) culture media were purchased from Gibco (Paisley, UK), and l-glutamine from Gibco and Lonza (Basel, Switzerland), penicillin and streptomycin from MP Biomedicals (Santa Ana, CA), Lonza, 10x trypsin/EDTA solution (5.0 g/l porcine trypsin and 2.0 g/l EDTA●4Na in 0.9% sodium chloride) from PAA Laboratories GmbH (Pasching, Austria), fetal calf serum (FCS) from PAA Laboratories and Biowest (Alkmaar, The Netherlands), and T75 cell culture flasks from Greiner-Bio One B.V. (Alphen a/d Rijn, The Netherlands). All buffers were prepared using Milli-Q water (mQ) generated from a Q-Gard 2 system (Millipore). Control Visucon-F plasma pool (citrated and 0.02 m Hepes buffered plasma pool from 20 healthy human donors) was obtained from Affinity Biologicals (Ancaster, Canada). The mRNA Capture kit was obtained from Roche and the reverse transcription system kit from Promega (Madison, WI).

##### Cells and Cell Culture

Human colorectal cancer cell lines (see Table I and Supplemental Table S1) were obtained from the Department of Surgery of the Leiden University Medical Center (LUMC), Leiden, The Netherlands, as well as the Department of Pathology of the VU University Medical Center (VUmc), Amsterdam, The Netherlands. Cells were cultured in Hepes-buffered RPMI 1640 culture medium containing l-glutamine and supplemented with penicillin (5000 IU/ml), streptomycin (5 mg/ml), and 10% (v/v) fetal calf serum (FCS) at the LUMC or in DMEM medium, supplemented with 10% (v/v) FCS and antibiotics, except for the KM12 cell line, which was grown in RPMI/10% FCS/antibiotics and l-glutamine at the VUmc. Cells were incubated at 37 °C with 5% CO_2_ in humidified air and cell culturing was performed up to a confluence of 80% under sterile conditions. For harvesting of the cells, medium was removed and adherent cells were washed twice with 1x PBS and trypsinized using 1x trypsin/EDTA solution in 1x PBS. To stop trypsin activity, medium in a ratio of 2:5 (trypsin/EDTA/medium; v/v) was added and cells were pelleted at 300 × *g* for 5 min. Cells were then resuspended in 3 ml 1x PBS and counted using the Countess^TM^ Automated Cell Counter (Invitrogen, Paisley, UK) based on tryptan blue staining. Cells were aliquoted to 2.0 × 10^6^ cells per ml of 1x PBS, and washed twice with 1 ml 1x PBS for 3 min at 1000 × *g*. The supernatant was removed and pellets stored at −20 °C until further use.

**Table I:** Overview of colorectal cancer cell lines

No.	Sample	Staging	Tumor site	Gender	Age
1	C10	2	Primary	Male	71
2	CaCo2	2	Primary	Male	72
3	Co115	3	Primary	Female	77
4	Colo205_VUmc	4	Ascitic fluid (metastatic site)^b^	Male	70
5	Colo320_VUmc	3	Primary	Female	58
6	DLD-1^a^	3	Primary	Male	45
7	HCT116	4	Primary	Male	48
7b	HCT116_VUmc	4	Primary	Male	48
8	HCT15_VUmc^a^	3	Primary	Male	
9	HCT8^a^	3	—	Male	67
10	HT29^c^	3	Primary	Female	44
10b	HT29_VUmc	3	Primary	Female	44
11	KM12_VUmc	2	Primary	—	—
12	LOVO	3	Lymph node metastasis	Male	56
13	LS174T_VUmc^d^	2	Primary	Female	58
14	LS-180^d^	2	Primary	Female	58
15	LS-411N	2	Primary	Male	32
16	RKO_VUmc	—	—	—	—
17	SW1116	1	Primary	Male	73
18	SW1398_VUmc	—	—	—	—
19	SW1463	3	Primary	Female	66
20	SW48	3	Primary	Female	82
21	SW480^e^	2	Primary	Male	51
21b	SW480_VUmc^e^	2	Primary	Male	51
22	SW620^e^	3	Lymph node metastasis	Male	51
23	SW948_VUmc	3	Primary	Female	81
24	T84	—	Lung metastasis	Male	72
25	WiDr^c^	—	Primary	Female	78

^a^ DNA profiling studies (57, 58) have shown that DLD-1, HCT-15, HCT-8 and HRT-18G share a single profile.

^b^ Treated with 5-fluorouracil for 4–6 weeks before collection.

^c^ Derivatives.

^d^ Variants/sister cell lines.

^e^ Same patient.

##### Release of N-glycans from Glycoprotein

Cell pellets (∼2 × 10^6^ cells) from two or three biological replicates of each colorectal cancer cell line (see Table I and Supplemental Table S1) were suspended in 100 μl mQ and sonicated in a water bath for 30 min. As control, 20 μl of human control Visucon-F plasma was used that was brought to 100 μl with mQ. Glycans were released using a PVDF-membrane based protocol adapted from Burnina *et al.* (23). Shortly, 0.25 × 10^6^ cells/well or 25 μl/well diluted human plasma in denaturation buffer (5.8 m GuHCl and 5 mm DTT), were loaded in quadruplicate onto preconditioned HTS 96-well plates with hydrophobic Immobilon-P PVDF membrane and incubated for 30 min at 60 °C in a moistured, sealed box as an incubation chamber within an oven. Plates were shaken for 5 min on a horizontal shaker prior to centrifugation (1 min, 500 × *g*). The wells were washed twice with 200 μl mQ with 2 min incubation steps on a horizontal shaker prior to centrifugation and once with 200 μl 100 mm NaHCO_3_ (1 min, 500 × *g*). For *N*-glycan release, 50 μl 100 mm NaHCO_3_ and 1 mU PNGase F (Roche) were added per well. Plates were placed into the incubation device and incubated for 3 h at 37 °C. Glycans were recovered into 96-well collection plates by centrifugation (2 min, 1000 × *g*); eventual residual solution was collected from the membrane.

##### Release of N-glycans from the Cell Surface

A subset of CRC cell lines were used to obtain the cell surface *N*-glycome in comparison to the *N*-glycosylation profile of the residual pellet using a protocol modified from Hamouda *et al.* (26). CRC cells were cultured as described above. Cells were harvested, pelleted, and aliquoted in Eppendorf tubes to 4 × 10^6^ cells in 1x PBS. Subsequently, cells were washed 2 × 10 min and 1 × 5 min at 300 × *g* with 500 μl 1x PBS. Next, cell pellets were carefully dissolved in 500 μl 1x PBS and 3.5 μl recombinant PNGase F (∼9 μg) were added and samples incubated for 30 min at 37 °C and 250 rpm in an Innova 43 incubator shaker (New Brunswick, Enfield, CT). Afterwards, samples were centrifuged 15 min at 500 × *g* and the supernatant containing the released glycans was collected. Supernatant and residual pellet were stored separately at −20 °C until further use. *N*-glycans from residual pellets were then released using the PVDF-membrane based protocol as described, but with 1 million cells per well and overnight PNGase F incubation.

##### Derivatization of N-glycans and Hydrophilic Interaction Liquid Chromatography (HILIC) Solid Phase Extraction Glycan Enrichment

Released *N*-glycans were derivatized by ethyl esterification adapted from Reiding *et al.* (24) allowing for discrimination of *N*-acetylneuraminic acid linkages (α2,3 *versus* α2,6). Briefly, 20 μl of released *N*-glycans from the total and residual cell pellets as well as the control samples were added to 100 μl of ethyl esterification reagent (0.25 m EDC and 0.25 m HOBt, 1:1 v/v). For the cell surface glycans, the supernatant was concentrated in a vacuum centrifuge to a volume of 20–30 μl, and 100 μl ethyl esterification reagent was added. Samples were incubated for 1 h at 37 °C. Subsequently, 100 μl ACN were added and the mixture was incubated at −20 °C for 15 min. Samples were brought to room temperature prior to glycan purification by hydrophilic interaction liquid chromatography (HILIC) solid phase extraction modified from a protocol described previously (27). For purification of pellet derived *N*-glycans, pipette tips of 20 μl volume were packed with a 4 mm piece of cotton thread (ca. 200 μg of cotton), equilibrated by pipetting 3 × 20 μl mQ water, followed by 3 × 20 μl 85% ACN. Samples were loaded by carefully pipetting up and down for 50 times. The tips were washed by pipetting 3 × 20 μl 85% ACN/1% TFA and 3 × 20 μl 85% ACN. *N*-glycans were eluted in 10 μl mQ water. For cell surface glycans, pipette tips of 200 μl volume were packed with cotton wool (ca 1000 μg) to prevent clogging through salts. The cotton HILIC purification was performed as described but with 150 μl pipetting volume, while elution was kept at 10 μl mQ water.

##### MALDI-TOF-MS Analysis

For mass spectrometric analysis, 5 μl of derivatized *N*-glycans were spotted onto an anchor chip MALDI target plate (Bruker Daltonics) and cocrystallized with 1 μl of 1 mg/ml superDHB in ACN/mQ (1/1, v/v) containing 1 mm NaOH. Samples were allowed to dry at room temperature and were then recrystallized with 0.5 μl 5 mg/ml superDHB in ACN/mQ (1/1, v/v) containing 1 mm NaOH. MALDI-TOF-MS spectra were acquired using an UltrafleXtremeTM mass spectrometer in the positive-ion reflector mode, controlled by FlexControl 3.4 software Build 119 (Bruker Daltonics). The instrument was calibrated using a Bruker peptide calibration kit. Spectra were obtained over a mass window of *m/z* 1000–5000 with ion suppression below *m/z* 900 for a total of 10,000 shots (1000 Hz laser frequency, 200 shots per raster spot during complete random walk). Tandem mass spectrometry (MALDI-TOF-MS/MS) was performed for structural elucidation via fragmentation in gas-off TOF/TOF mode.

##### Data Processing of MALDI-TOF-MS Spectra

A mean average spectrum over all the total cell line sample spectra was generated using an in-house developed script in Python 2.7.3 (Python Software Foundation; http://docs.python.org/py3k/reference/index.html). The average spectrum was internally recalibrated using glycan peaks of known composition (Supplemental Table S2), smoothed (Savitzky Golay algorithm, peak width: *m/z* 0.06, four cycles), and baseline-corrected (Tophat algorithm) using FlexAnalysis Software (Version 3.3; Bruker Daltonics). Peaks of signal-to-noise > 2 were picked, manually revised, and analyzed in GlycoWorkbench 2.1 stable build 146 (http://www.eurocarbdb.org/) using the Glyco-Peakfinder tool (http://www.eurocarbdb.org/ms-tools/) for generation of a glycan compositions list. Using MassyTools version 0.1.5.1, which is a novel in-house software developed for automated data processing, the resulting glycan peak list generated based on the average spectrum was used for targeted data extraction of the area under the curve for each of the CRC cell line *N*-glycomic profiles (28). Within this software, background was determined dynamically and subtracted from intensities of all isotopic peaks prior to calibration of each spectrum and targeted data extraction. Several quality parameters are calculated in order to assess the quality of each individual spectrum as well as picked glycan peaks and allow for good quality data selection. Based on quality parameters, the composition list was reviewed, and only the glycans exhibiting a correct isotopic peak pattern and an average signal noise per cell line above 6 in at least 50% of the spectra of a specific cell line (including both technical and biological replicates) were used for final data extraction. Selected glycan compositions were confirmed by MS/MS. The final peak list as well as MS/MS data are listed in Supplemental Table S3. Further, glycan profiles had to pass at least two out of three quality criteria for inclusion: (i) total intensity > 1 × 10^5^, (ii) fraction of analyte area with signal noise > 6 is more than 80%, (iii) fraction of glycan analyte intensity in total spectrum intensity > 40%. The area-under-the-curve values were rescaled to a total relative intensity of 100% for each spectrum.

For the comparison of cell surface and residual pellet *N*-glycomes, a condensed peak list was used due to the slightly lower spectral quality which resulted in the detection of less analytes.

##### Data and Statistical Analysis

Averages, standard deviations, and relative standard deviations were calculated for all biological replicates of the cell lines (Supplemental Table S4). Preprocessed, selected, and rescaled total cell *N*-glycome data was imported into SIMCA software Version 13.0 (Umetrics AB, Umea, Sweden), and a principle component analysis (PCA) was performed to reveal outliers and batch effects and to study the effects of possible confounders. A PCA displays the variation in the data on new vectors (principle components) and makes it possible to visualize the differences in the data in a two-dimensional way. The PCA is an unsupervised model that finds “natural” variation in the data without over-fitting. The observations (= cell line samples) are displayed in the score plots, while the variables (= relative intensity values for each glycan) are displayed in the loading plots. The glycans located at the outskirts of the loading plot are those contributing most to the displayed principle components. The samples located on the outskirts of the score plot are those showing a particularly large deviation (variation) from the other samples. In addition, the particular locations of data points in score and loading plots are associated. For example, samples with particularly high intensities of glycans located in the top left of the loading plot will locate in the top left of the corresponding score plot. Furthermore, we classified the glycans in traits such as levels of fucosylation and sialylation, for which later also relative abundances were calculated (see below). We used these traits to color the variables in the loading plots to find possible contributions of glycan traits to principle components.

To validate observed trends in PCA, averages per cell type (biological replicates) were used to calculate glycan traits (calculations see Table II). Relative intensities were first summed according to *N*-glycan types (pauci-mannose, high-mannose, hybrid, and complex type). Data were then rescaled to 100% excluding high-mannose-type glycans to prevent influences of possible high-mannose-type intracellular precursors. Then, additional glycan-derived traits for complex- and hybrid-type glycans were calculated (mono-fucosylation, multi-fucosylation, α2,6- and α2,3-sialylation, ratio Hex *versus* HexNAc, and number of antennae) and compared (Supplemental Table S5). Mann–Whitney and Kruskal–Wallis with Tukey's posttest with significance level α = 0.05 were performed in GraphPad Prism Version 5.04 (GraphPad Software, Inc., La Jolla, CA) to explore differences of glycan traits with cell line characteristics such as stage of the original tumor, tumor site (primary *versus* metastasis), and differentiation.

##### AA-Labeling and LC-ESI-ion trap-MS/MS

Released glycans from technical replicates of total cell pellets were pooled per cell line and 20 μl sample incubated with 40 μl AA-labeling solution (48 mg/ml AA in DMSO/15% glacial acetic acid and 1 m 2-picoline borane in DMSO, 1:1, v/v) for 2 h at 60 °C (29). Samples were cooled down to room temperature, brought to 85% ACN, and purified by cotton-thread HILIC solid phase extraction as described before but with elution in 5 μl mQ water. AA-labeled glycans were transferred to glass vials with insert and 0.5 μl of sample was injected into a nano-RP-LC-ESI-ion trap-MS(/MS) system for glycan fragmentation analysis. Within the Ultimate 3000 RSLCnano system (Thermo Scientific, Sunnyvale, CA), samples were first concentrated onto a trap column (Acclaim PepMap100 C18 column, 100 μm × 2 cm, C18 particle size 5 μm, pore size 100 Å, Thermo Scientific) prior to separation on an Acclaim PepMap RSLC nano-column (75 μm × 15 cm, C18 particle size 2 μm, pore size 100 Å, Thermo Scientific). A flow rate of 700 nl/min was applied in a multistep linear gradient (t = 0–5 min, c(B) = 3%; t = 35 min, c(B) = 27%; t = 40–45 min, c(B) = 70%; t = 46–58 min, c(B) = 3% with 0.1% formic acid in water as solvent A, and 95% ACN and 5% water as solvent B). The separation was also monitored by UV absorption at 215 nm.

The LC-system was coupled to a CaptiveSpray nanoBooster (Bruker Daltonics) for mass spectrometric MS/MS analysis on an AmazonSpeed ion trap (Bruker Daltonics) using a captive spray for ionization of samples in the positive ion mode. For the electrospray (1300 V), fused-silica capillaries with an internal diameter of 20 μm were used. Solvent evaporation was achieved at 220 °C with an ACN enriched nitrogen nebulizer gas at a pressure of 0.2 bar. Tandem MS was performed automated on the seven highest precursors per MS with ion detection from *m/z* 100 to 3000. Fragmentation spectra were analyzed using Data Analysis 4.2 Build 387 (Bruker Daltonics). Analysis on AA-labeled glycans was exclusively used for structural elucidation (Supplemental Table S3) and targeted search for indicative fragment ions of blood group antigens (Supplemental Table S6).

##### RNA Isolation and cDNA Synthesis

For mRNA analysis, 1 million of the cultured cells were transferred to RNase-Free Eppendorf tubes, centrifuged 5 min at 300 × *g*, pelleted, and lysed in 500 μl of lysis buffer. Lysated cells were stored at −80 °C until mRNA was specifically isolated by capture of poly(A+) RNA in streptavidin-coated tubes using a mRNA Capture kit. cDNA was synthesized using a Reverse Transcription System kit following the manufacturer's guidelines. Lysates were incubated with biotin-labeled oligo(dT)20 for 5 min at 37 °C and then 50 μl of the mix was transferred to streptavidin-coated tubes and incubated for 5 min at 37 °C. After washing three times with 250 μl of washing buffer, 30 μl of the reverse transcription mix (5 mm MgCl_2_, 1× reverse transcription buffer, 1 mm dNTP, 0.4 U of recombinant RNasin RNase inhibitor, 0.4 U of reverse transcriptase, 0.5 μg of random hexamers in nuclease-free water) were added to the tubes and incubated for 10 min at room temperature followed by 45 min at 42 °C. To inactivate avian myeloblastosis virus (AMV) reverse transcriptase and separate mRNA from the streptavidin-biotin complex, samples were heated at 99 °C for 5 min, transferred to microcentrifuge tubes, and incubated in ice for 5 min, diluted 1:2 (v/v) in nuclease-free water, and stored at −20 °C until analysis.

##### Real-Time PCR

Oligonucleotides for 17 glycosyltransferases (GTs) were designed by using the computer software Primer Express 2.0 (Applied Biosystems, Foster City, CA), synthesized by Invitrogen Life Technologies (Breda, The Netherlands), and are published elsewhere (30). PCRs were performed with the SYBR Green method in an ABI 7900HT sequence detection system (Applied Biosystems). The reactions were set on a 96-well-plate by mixing 4 μl of the two times concentrated SYBR Green Master Mix (Applied Biosystems) with 2 μl of a oligonucleotide solution containing 5 nm/μl of both oligonucleotides and 2 μl of a cDNA solution corresponding to 1:100 (v/v) of the cDNA synthesis product. The thermal profile for all the reactions was 2 min at 50 °C, followed by 10 min at 95 °C and then 40 cycles of 15 s at 95 °C and 1 min at 60 °C. The housekeeping gene glyceraldehyde-3-phosphate dehydrogenase was used as endogenous reference (31).

##### Glycosyltransferase mRNA Data and Statistical Analysis

To calculate the relative abundance of the genes, the formula 100 × 2 (Ct glyceraldehyde-3-phosphate dehydrogenase -Ct glycosyltransferase) was used, where Ct is the cycle threshold. In this formula, the Ct value is defined as the number of PCR cycles at which the SYBR-green fluorescent signal exceeds the threshold of 0.2 relative units (32). Averages were calculated for nine technical replicates and the three highest and lowest values were colored in green and red, respectively (Supplemental Table S7). In order to improve comparability, mRNA data was as well LOG2 transformed. To assess the quality and discriminative power, GT mRNA data were imported into SIMCA software and a PCA model was built as described above. The nine technical replicates were used as individual cross-validation groups. The median across the included cell lines was calculated and GT mRNA data was compared with relative abundances of corresponding glycan traits based on the total *N*-glycome. Both derived traits based on MS data as well as GT gene expression data were imported into GraphPad Prism (GraphPad Software), and a linear regression model was calculated.

CDX1- and villin expression data were based on (33–35) while coexpression data with Pearson correlations was retrieved from the Cancer Cell Line Encyclopedia (Supplemental Table S8) via the database http://www.cbioportal.org/ (36, 37).

## RESULTS

### 

#### 

##### High-Throughput Method for Cell Line N-glycosylation Profiling

We established a high-sensitivity method for MALDI-TOF-MS profiling of *N*-glycans from cell lines. Cellular (glyco-) proteins were solubilized in chaotropic agents and applied to PVDF-membranes in a 96-well format (23). *N*-glycans were released from adsorbed proteins by PNGase F and derivatized by ethyl esterification to achieve stabilization of sialic acids as well as differentiation of sialic acid α2,3- and α2,6-linkages (24). Glycans were purified using cotton-HILIC micro-solid phase extraction (27) and analyzed by MALDI-TOF-MS (see workflow in Supplemental Fig. S1). This novel workflow showed high sensitivity and allowed the robust acquisition of high-quality *N*-glycan profiles from 250,000 cells. The total cell *N*-glycan profiling workflow was applied to analyze 25 CRC cell lines in multiple biological and technical replicates per cell line, in order to be able to discern biological and technical variation. Applying an unsupervised principle component analysis (PCA) revealed only little variation between replicates (technical as well as biological) of each cell line as seen by close clustering of scores (Supplemental Figs. S2*A* and 2*B*). The dataset was further analyzed for possible batch effects. PCA scores were colored according to batches (Supplemental Fig. S2*C*), and technical replicates distributed on different plates as well as different sample preparations were compared. Based on this comparison, batch effects were found to be minor and no correction was applied. Relative intensities for all glycans in the biological replicates of the cell lines with standard deviation and relative standard deviation are given in Supplemental Table S4. Relative standard deviations were below 20% for peaks with relative intensities above 5%, with exception for cell line C10, showing slightly higher variation. Although this method already provided a robust data acquisition, glycan traits were calculated by summing up relative intensities of glycan peaks corresponding to a certain glycan class (see Table II) to further increase the robustness of the data analysis.

**Table II:** Formulas for derived trait calculations

Derived traits	Calculation (relative intensity total 100%)
Glycan type:	
High-mannose	Σ(Hex4–10HexNAc2)
Pauci-mannosidic	Σ(Hex1–3HexNAc2)
Hybrid	Σ(Hex-HexNAc)>2)
Complex	Σ(Hex-HexNAc)≤1)
Derived traits	Calculation (relative intensity complex and hybrid 100%)
Antennarity:	
Mono-	Σ(HexNAc≤3)
Di-	Σ(Hex = 5) and (HexNAc = 4)
Di/tri-	Σ(Hex≤5) and (HexNAc = 5)
Tri-	Σ(Hex = 6) and (HexNAc = 5)
Tri/tetra-	Σ(Hex≤6) and (HexNAc = 6)
Tetra-	Σ(Hex = 7) and (HexNAc = 6)
Tetra/poly-	Σ(Hex≤7) and (HexNAc = 7)
Poly-	Σ(Hex>7) and (HexNAc>6)
Fucosylation:	
Mono-fucosylation	Σ(Fuc = 1)
Multi-fucosylation	Σ(Fuc>1)
Sialylation:	
α2,6-sialylation (pure)	Σ(ENeuAc≥1) and (LNeuAc = 0)
α2,3-sialylation (pure)	Σ(LNeuAc≥1) and (ENeuAc = 0)
Mixed sialylation	Σ(LNeuAc≥1) + (ENeuAc≥1)
Hex/HexNAc ratio:	
Hex>HexNAc	Σ((Hex-HexNAc)≥1)
Hex = HexNAc	Σ((Hex-HexNAc) = 0)
Hex<HexNAc	Σ((Hex-HexNAc)<0)

Hexose = Hex, H; *N-*acetylhexosamine = HexNAc, N; α2,6-*N-*acetylneuraminic acid = ENeuAc, E; α2,3-*N-*acetylneuraminic acid = LNeuAc, L; fucose = dHex, Fuc, F; * The presence of poly-LacNAc structures is exclusively considered in poly-antennarity.

##### N-glycosylation Profiling of 25 Colorectal Cancer Cell Lines

**Fig. 1.**
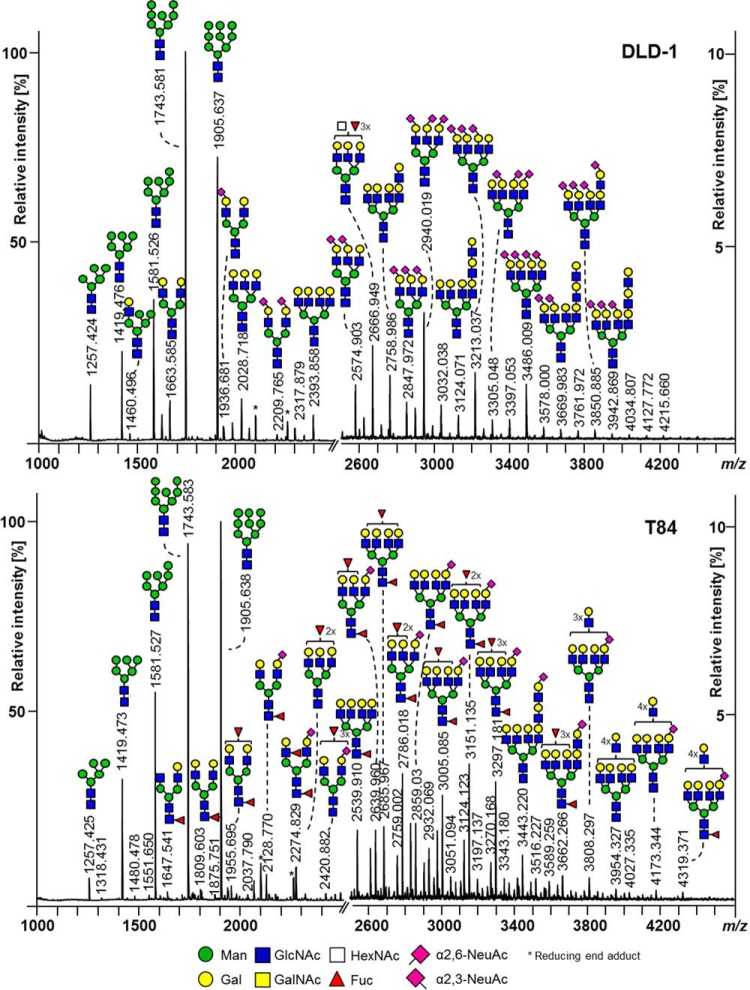
**MALDI-TOF-MS N-glycome spectra of two exemplary colorectal cancer (CRC) cell lines:** (*A*) DLD-1, and (*B*) T84. *N*-glycans were detected as sodium adduct ions [M+Na]^+^ over a mass range of *m/z* 1000–4500. Spectra show relative intensities with zoom on the mass range *m/z* 2500–4500. Major glycan peaks are annotated and represent compositions. The presence of structural isomers cannot be excluded. Linkage positions of sialic acid residues are indicated by differing angles.

**Fig. 2.**
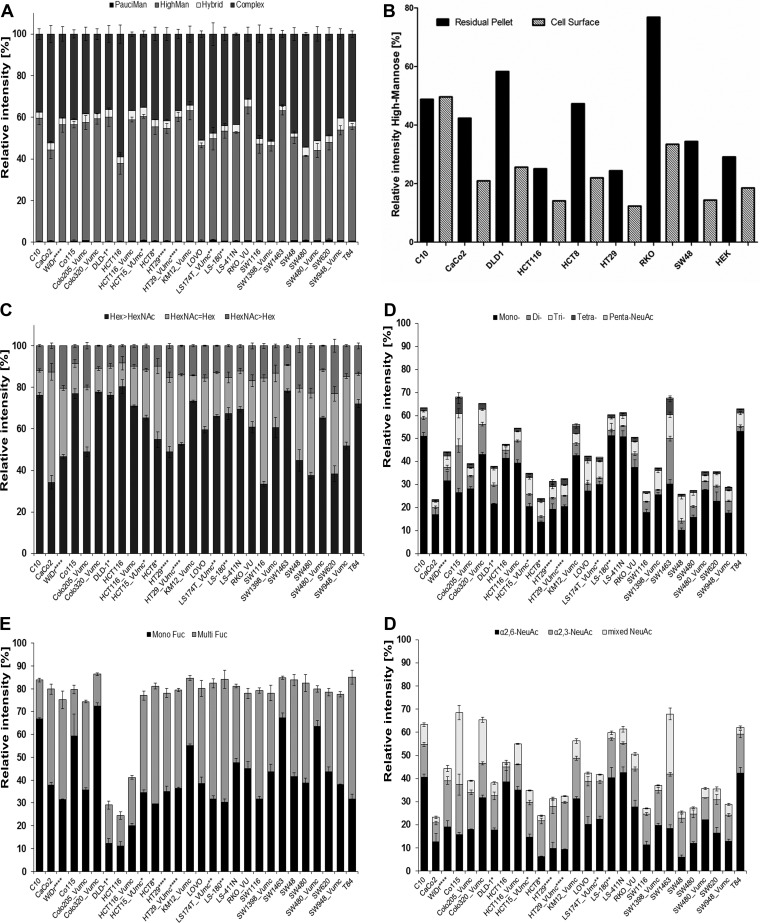
**Relative quantification of derived glycan traits.** Summed relative intensities from MALDI-TOF-MS analysis according to glycan classes: (*A*) *N*-glycan types; (*B*) high-mannose *N*-glycan content on the cell surface of CRC cell lines *versus* the content in the residual pellets; (*C*) hexose (Hex)/*N*-acetylhexosamines (HexNAc) ratios: Equal amounts of Hex and HexNAc of complex- and hybrid-type *N*-glycans (Hex = HexNAc) indicates presence of either bisection or terminal HexNAc, whereas HexNAc>Hex ratio points additionally to the presence of terminal HexNAc in form of *e.g.* LacdiNAc structures; (*D*) antennary of complex- and hybrid-type *N*-glycans; (*E*) fucosylation distinguishing mono-fucosylated (MonoFuc, one fucose) and multi-fucosylated (MultiFuc, 2–5 fucoses) on complex- and hybrid-type *N*-glycans; (*F*) purely α2,6- and α2,3-sialylated complex- and hybrid-type *N*-glycans as well as glycans with mixed sialylation. Error bars display the standard deviation between 2–3 biological replicates. Relative intensities were rescaled to 100% for calculation of traits corresponding to complex- and hybrid-type *N*-glycans (*A*, *C-F*). *DNA profiling studies (57, 58) have shown that DLD-1, HCT-15, HCT-8 and HRT-18G share a single profile; ** variants/sister cell lines; *** same patient;**** derivatives.

**Fig. 3.**
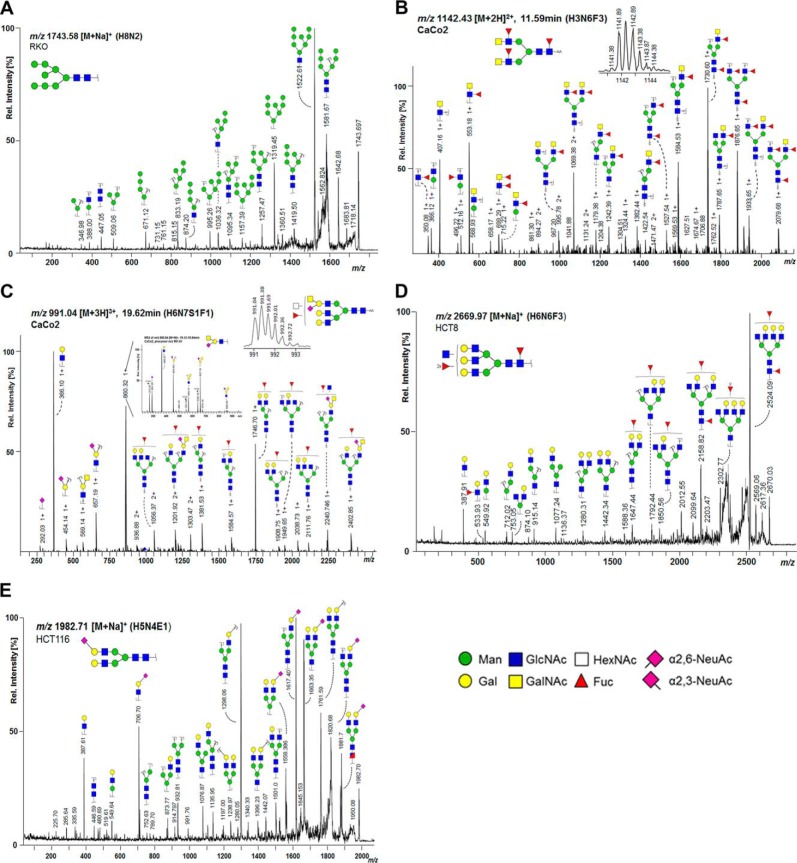
**MS/MS analysis of *N-*glycans.** (*A*) MALDI-TOF/TOF-MS/MS spectrum of *m/z* 1743.58 [M+Na]^+^ from CRC cell line RKO confirming the high-mannose composition Hex8HexNAc2; (*B*) LC-ESI-MS/MS spectrum of *m/z* 1141.94 [M+2H]^2+^ from cell line CaCo2 confirms the composition Hex3HexNAc6dHex3 comprising two LacdiNAc (GalNAcβ1–4GlcNAcβ1-) antennae as indicated by the fragment ion *m/z* 407.16 [HexNAc2]^+^ and its fucosylated variant with *m/z* 553.18 [HexNAc2dHex1]^+^. The fragment ion *m/z* 699.29 [HexNAc2dHex2]^+^ may be the result of a fucose re-arrangement (59); (*C*) LC-ESI-MS/MS/MS spectrum of 991.04 [M+2H]^3+^ confirming the composition Hex6HexNAc7NeuAc1dHex1 in CaCo2 cells showing a specific fragment ion at *m/z* 860.64 [M+H]^+^ (Hex1HexNAc2NeuAc1). MS3 (zoom) of the fragment ion *m/z* 860.64 [M+H]^+^ from cell line CaCo2 confirms the presence of Sda-antigen (NeuAcα2–3[GalNAcβ1–4]Galβ1–4GlcNAc-R); (*D*) MALDI-TOF/TOF-MS/MS spectrum of *m/z* 2669.97 [M+Na]^+^ from CRC cell line HCT8 confirming the tri-fucosylated *N*-glycan with the composition Hex6HexNAc6dHex3 and an additional agalactosylated antenna or bisected GlcNAc; (*E*) MALDI-TOF/TOF-MS/MS spectrum of *m/z* 1982.71 [M+Na]^+^ from CRC cell line HCT116 confirming the composition of the complex type *N*-glycan Hex5HexNAc4α2,6NeuAc1 with fragment ion *m/*z 706.70 [M+Na]^+^ (Hex1HexNAc1α2,6NeuAc1). The presence of structural isomers cannot be excluded. Annotation was performed using GlycoWorkbench 2.1 stable build 146 (http://www.eurocarbdb.org/).

This sensitive and robust *N*-glycan profiling method was used to characterize the *N*-glycosylation of 25 CRC cell lines. Exemplary *N*-glycan profile spectra of two cell lines are shown in Fig. 1, while a complete set of *N*-glycan profiles is provided in Supplemental Fig. S3. Calculated traits are depicted in Fig. 2 and Supplemental Table S5. The *N*-glycan profiles of almost all CRC cell lines were dominated by high-mannose-type glycans (37.5% to 64.3%, Ø 53.0%, Figs. 1 and 2*A*; for structural evidence see Fig. 3*A*). The only exception was the cell line HCT116 which also showed a high abundance of complex type glycans (58.9%). Complex type glycans could be detected up to *m/z* 4500 and their abundance varied considerably among the different cell types (31.3% to 58.9%, Ø 43.1%, Fig. 2*A*). The high abundance of high-mannose-type glycans may reflect a vast contribution of intracellular precursors, especially indicated by the glycan Man9HexNAc2Glc1 (*m/z* 2067.69), which led to the decision to exclude high-mannose-type glycans to calculate the derived glycosylation traits related to complex type *N*-glycans (*e.g.* Hex/HexNAc ratio, sialylation levels). To prove, however, that high-mannose-type glycans are indeed also present on the cell surface, *N*-glycans were shaved of the plasma membrane in a 30-min incubation step of living cells in the presence of high concentrations of recombinant PNGase F. The relative abundance of high-mannose-type *N*-glycans on the cell surface with respect to the residual pellet is given in Fig. 2*B*, showing that high-mannose-type *N*-glycans are expressed on plasma membrane proteins but to a lower extent as compared with their presence as intracellular precursor. The cell line C10 is an exception and shows similar levels of high-mannose-type glycans on the cell surface and the residual pellet. An exemplary MS spectrum comparing the cell surface *N*-glycome of CaCo2 cells with the residual pellet containing intracellular precursors is shown in Supplemental Fig. S4.

**Fig. 4.**
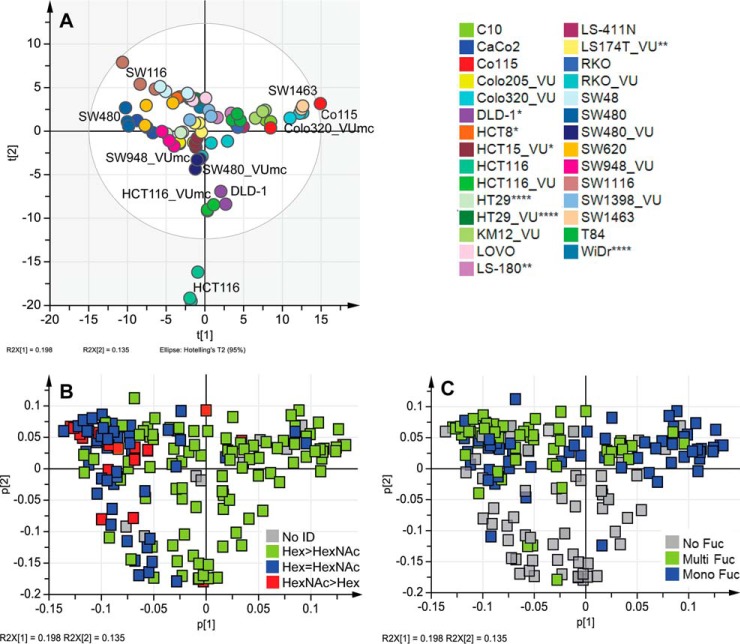
**Principal component analysis (PCA).** Averages of biological replicates per glycan and cell line were unit-variance scaled and used for multivariate data analysis in SIMCA V13 (Umetrics, Sweden). The PCA model resulted in 13 principal components (PCs) explaining 84.4% variation (R^2^X(cum)) within the data and a good prediction power Q^2^(cum) of 65.4%. Cross validation was performed on technical replicates distributed into six groups. (*A*) PCA score plot of PC1 (19.8%) against PC2 (13.5%) colored according to cell lines; (*B* and *C*) loading plot of PCA model displaying PC1 (19.8%) against PC2 (13.5%) colored according specific glycan features: (*B*) ratio between the number of hexoses (Hex) and the number of *N*-acetylhexosamines (HexNAc) with Hex>HexNAc representing fully galactosylated antennae, while HexNAc≥Hex indicates terminal HexNAc with indications for bisection (HexNAc = Hex) and LacdiNAc epitopes (GalNAcβ1, 4GlcNAcβ1-; HexNAc>Hex) and (*C*) fucosylation distinguishing mono-fucosylated (Mono Fuc) *versus* multi-fucosylated (Multi Fuc) *versus* non-fucosylated (No ID) *N*-glycans. *DNA profiling studies (57, 58) have shown that DLD-1, HCT-15, HCT-8 and HRT-18G share a single profile; ** variants/sister cell lines; *** same patient;**** derivatives.

For further data analysis, a PCA model based on biological replicates (averaged technical replicates) was generated using SIMCA software. This resulted in a model explaining 84.4% (R^2^Xcum) of the data with 13 principal components (PCs) featuring a good prediction power of 65.4% (Q^2^cum). Coloring the scores according to cell lines showed clear clustering based on the cell type as shown in Fig. 4*A*, indicating robust glycosylation features shared by biological and technical replicates. Cell line samples clustered within the Hotelling's T 95% with exception of HCT116, which seems to differ vastly from other tested cell lines. Coloring the loading plots, which represent each glycan variable, according to glycan classes helped to exploit which differences in glycosylation drove the separation in the PCA model. Trends observed in PCA models were then compared with relative abundancies of glycan classes by derived trait calculations based on the total cellular *N*-glycome.

##### Hex/HexNAc Ratio

PC1 (19.8%) appeared to reflect glycans differing in their hexose (Hex)/*N*-acetylhexosamine (HexNAc) ratio (Fig. 4*B*). Equal amounts of HexNAc and Hex indicate the presence of terminal *N*-acetylglucosamine (GlcNAc) or bisecting GlcNAc, while higher amounts of HexNAc than Hex may additionally point to the presence of terminal *N*-acetylgalactosamines (GalNAc) in *e.g.* LacdiNAc structures (GalNAcβ1–4GlcNAcβ1-). PC3 (12.3%) appeared to likewise separate on Hex>HexNAc *versus* Hex<HexNAc, while PC4 (7.2%) refined the separation into terminal/bisecting HexNAc (upper half) and glycans with potential LacdiNAc epitopes (*lower half*; Supplemental Figs. S5*A* and S5*B*). The presence of LacdiNAc epitopes was supported by the detection, albeit with low intensity, of a fragment ion at *m/z* 429 (HexNAc2 [M+Na]^+^) in MALDI-TOF/TOF-MS/MS spectra of some glycans such as for instance Hex3HexNAc5dHex1 with *m/z* 1688.61 [M+Na]^+^ (Supplemental Table S3). To further confirm the presence of LacdiNAc epitopes, glycan samples were labeled by 2-AA and analyzed by RP-LC-ESI-MS/MS. As shown in Fig. 3*B* and described in Supplemental Table S3, the detection of the fragment ion at *m/z* 407 (HexNAc2 [M+H]^+^) and its fucosylated variant at *m/z* 553 (HexNAc2dHex1 [M+H]^+^) in the fragmentation spectrum of the glycan Hex3HexNAc6dHex3-AA unambiguously demonstrated that this glycan carries a LacdiNAc motif. Additionally, the sialylated counterpart at *m/z* 698 (HexNAc2NeuAc1 [M+H]^+^) was found in some glycans (Supplemental Table S3). With 20.0% to 22.7%, SW480, SW620, SW48, Colo205_VUmc, and WiDr showed highest levels of *N*-glycans with HexNAc>Hex, while CRC cell lines SW1463, DLD-1, HCT15_VUMC, and HCT8 (the latter three share DNA profile) contained low levels (8.3–9.9%, Fig. 2*C*, Supplemental Table S5). In CaCo2 and SW116 cells, 53.2% and 51.2% of the glycans, respectively, contained the same amount of Hex and HexNAc, which potentially reflected the presence of glycans with terminal or bisecting HexNAc (Fig. 2*C*, Supplemental Table S5). For SW1116 and CaCo2, the presence of bisection was confirmed in RP-LC-ESI-MS/MS on AA-labeled glycans with the indicative fragment ion at *m/z* 1057 [M+H]^+^ (Hex1HexNAc3dHex1-AA; Supplemental Table S3). For CaCo2 cells, additionally a specific epitope was found in RP-LC-ESI-MS/MS analysis, consisting of NeuAc[GalNAc]Gal-GlcNAc-R (Fig. 3*C*). The characteristic fragment ion *m/z* 860 [M+H]^+^ was confirmed by MS3 (see Fig. 3*C*) and pointed to the presence of the Sda antigen.

##### Antennarity

The glycan antennarity is reflected in PC5 (6.7%) showing separation of cell lines expressing higher antennary glycans clustering on the left and lower antennary glycans on the right (Supplemental Figs. S5*C* and 5*D*). Poly-antennarity with more than four LacNAc units indicates the presence of LacNAc repeats (-3Galβ1–4GlcNAcβ1-). However, LacNAc repeats can also be present on di-, tri-, and tetra-antennary glycans, which could not be reflected in the trait calculations. MS data revealed the abundance of poly-antennary glycans in cell lines SW480 (21.1%), SW1116 (20.1%), SW48 (19.3%), and SW620 (18.6%) (Fig. 2*D*, Supplemental Table S5), which locate together with DLD-1, HCT116, and T84 on the left side of the score plot (= high antennarity; Supplemental Fig. S5*C*). The highest percentage of tri/tetra-antennary glycans in MS data showed cell line Caco2 (67.2%), followed by SW1398_VUmc (65.6%), Lovo (63.8%), SW48 (62.9%), and WiDr (61.5%; Fig. 2*D*, Supplemental Table S5), which cluster all around the intersection of PC1 according to the tri-/tetra-antennary glycans in the loading plot (Supplemental Figs. S5*C*and 5*D*). The lowest abundance of tri/tetra-antennary glycans was found in SW1463 (45.9%) and Co115 (48.1%; Fig. 2*D*, Supplemental Table S5). In accordance with their location in the PCA plots, SW1463 (30.8%), Co115 (29.0%), HCT116_VUmc (28.3%), and Colo320_VUmc (26.6%) exhibit the highest abundances of di-antennary glycans, while the highest levels of mono-antennary *N*-glycans were found in HCT15_VUmc (4.9%), SW948_VUmc (4.4%), HCT116_VUmc (4.3%), RKO_VUmc (4.3%), Colo205_VUmc (4.1%), and SW480_VUmc (3.8%) (Fig. 2*D*, Supplemental Table S5, Supplemental Figs. S5*C*and 5*D*). In contrast, CaCo2 is as well located on the right of the PCA score plot but is characterized by high levels of tri-/tetra-antennary glycans.

##### Fucosylation

While PC1, 3, and 4 appeared to be linked to antennarity and terminal HexNAc residues, PC2 (13.5%) was found to reflect fucosylation, separating fucosylated (upper half) from non-fucosylated glycans (lower half; Fig. 4*C*). Accordingly, score plots of PC1 *versus* PC2 (Fig. 4*A*) show HCT116 and DLD-1 located apart from the other cell lines in the lower half, in line with the low relative quantities of fucosylated glycans in these cell lines (Fig. 2*E*). Relative quantification of complex type glycans reveals only 24.4%,29.1%, and 41.2% fucosylation in HCT116, DLD-1, and HCT116_VUmc, respectively; whereas all other investigated CRC cell lines contain 74.4% to 86.4% fucosylated *N*-glycans (Fig. 2*E*, Supplemental Table S5). Cell line HCT116 has a mutation in the fucosylation pathway and is therefore expected to have low fucosylation. Splitting the total fucosylation in mono-fucosylation (one fucose) and multi-fucosylation (2–5 fucoses, indicative for the presence of Lewis-type antigens) revealed mono-fucosylation to be most abundant in Colo320_VUmc cells (72.4%), followed by SW1463 (67.3%), C10 (66.8%), and Co115 (59.4%). MS/MS spectra give evidence of core-fucosylation in many of the *N*-glycans, with the loss of the reducing end GlcNAc only occurring after the loss of fucose (Supplemental Fig. S3*D*, Supplemental Table S3). Nevertheless, mono-fucosylation can also occur on antenna and is not a reliable measure of core-fucosylated glycans. LS180 cells showed highest multi-fucosylation, based on relative quantification of MS data (53.7%), followed by cell lines T84 (53.4%), HCT8 (51.8%), LS174T (50.7%), and SW1116 (47.5%; Fig. 2*E*, Supplemental Table S5). The presence of antenna fucosylation is indicated by MS/MS data (Supplemental Table S3). Furthermore, antenna fucosylation with indications of the presence of blood group antigens was found (Supplemental Table S6) which is in accordance with literature information of the expression of blood group antigens in some of the cell lines (Supplemental Table S1). In cell line SW48 (blood type AB), fragment ions indicative of the presence of blood group A antigen (GalNAc-Gal-(Fuc)-GlcNAc-) at *m/z* 308.88 [Hex1dHex1+H]^+^, *m/z* 715.28 [Hex1HexNAc2dHex1]+, and *m/z* 861.31 [Hex1HexNAc2dHex2]^+^ as well as blood group antigen B (Gal-Gal-(Fuc)-GlcNAc-) at *m/z* 674.20 [Hex2HexNAc1dHex1]^+^ were found in RP-LC-ESI-MS/MS analysis of AA-labeled *N*-glycans (Supplemental Fig. S6). The presence of blood group A antigens further contributes to the trait Hex/HexNAc ratio. However, abundances of blood group antigens were rather low on *N*-glycans and expression of these antigens might be higher on other glycan and glycoconjugates such as *O*-glycans and glycosphingolipids.

##### Sialylation

PC6 (5.5%) showed separation according to sialylation with α2,6-sialylated glycans clustering in the upper half and α2,3-sialylated glycans in the lower half, respectively (Supplemental Figs. S5*C* and 5*E*). Relative abundances of α2,6-NeuAc containing *N*-glycans (pure and mixed) based on MS data showed the highest levels with 50.5% in Colo320_VUmc, 49.0% in C10, 48.7% in LS-411N, 45.2% in T84, and 42.9% in LS-180, while levels are lowest in HCT8 with 8.5% and SW48 with 8.8%, which is in accordance to their location in the PCA score plot (top half) (Fig. 2*F*, Supplemental Table S5, Supplemental Figs. S5*C*and 5*E*). The presence of α2,6-NeuAc is indicated by +319.13 Da in the profile spectra and was confirmed by tandem MS via marker ions of *m/z* 341.64 [M+Na]^+^ (α2,6NeuAc1) and *m/z* 706.71 [M+Na]^+^ (Hex1HexNAc1α2,6NeuAc1), as shown for the *N*-glycan Hex5HexNAc4α2,6NeuAc1 with *m/z* 1982.71 [M+Na]^+^ (Fig. 3*E*).

The presence of lactonized α2,3-NeuAc is characterized by +273.08 Da in the profiling spectra as well as by the tandem MS marker ion *m/z* 475.08 (Hex1α2,3NeuAc1; Supplemental Table S3). Abundance of α2,3-sialylated glycans (pure and mixed) is highest in Co115 (52.6%; Fig 2*F*, Supplemental Table S5). Also, SW1463 cells showed a high percentage of α2,3-linked sialic acid containing glycans (49.5%), while the lowest levels detected by MS were in HCT116 (8.6%) and CaCo2 (10.7%; Fig 2F, Supplemental Table S5). In some cell lines, as HT29, WiDr, SW48, T84, and Lovo, high expression of α2,3-sialylation (16.8% to 20.4%) was accompanied by high levels of multi-fucosylation (42.4% to 53.4%), which is indicative for the expected expression of antennary sialyl Lewis antigens (Figs. 2*E* and 2*F*). The presence of sialyl Lewis antigens was confirmed by RP-ESI-LC-MS/MS analysis on AA-labeled *N*-glycans, in which the diagnostic fragment ion at *m/z* 803.29 [Hex1HexNAc1NeuAc1dHex1]^+^ was found in several cell lines (Supplemental Table S6).

Overall, observations in the PCA plots could majorly be confirmed by relative quantification using derived traits, showing a vast difference between the cell lines. Furthermore, the coloring of loadings according to glycan classes was found to be a promising way to explore the data and to find the discriminators contributing to each principal component.

##### Comparison with N-glycans Derived from Tissues

The aim of this study was to further evaluate the potential of the studied CRC cell lines as model system. We therefore compared the *N*-glycan profiles derived from CRC cell lines (Supplemental Fig. 3 and Supplemental Table S4) with the data obtained for CRC-tumor and control tissues derived *N*-glycans by Balog *et al.* (10) (Supplemental Table S3). Profiles were further compared with recently published data on colorectal adenoma and carcinoma tissues (11), and most of the complex type glycans found in the cell lines were also present in tissues. Furthermore, typical cancer antigens like (sialyl) Lewis epitopes were preserved in the CRC cell lines. Also, the Sda-antigen that we found in CaCo2 cells was described to be expressed in the human gastrointestinal tract, while its expression is decreasing during CRC progression (38). The major difference between CRC cell line and tissue *N*-glycan profiles is the dominance of high-mannose-type glycans in most of the cells, which is partly due to intracellular precursors. Notably, high-mannose-type *N*-glycans were also present in CRC tissues (10, 11) and on the cell surface of CRC cell lines (Fig. 2*B*, Supplemental Fig. S4) but in lower relative abundance than in the total cell line profiles determined in this study.

##### Glycan Trait Associations with Stage and Differentiation

**Fig. 5.**
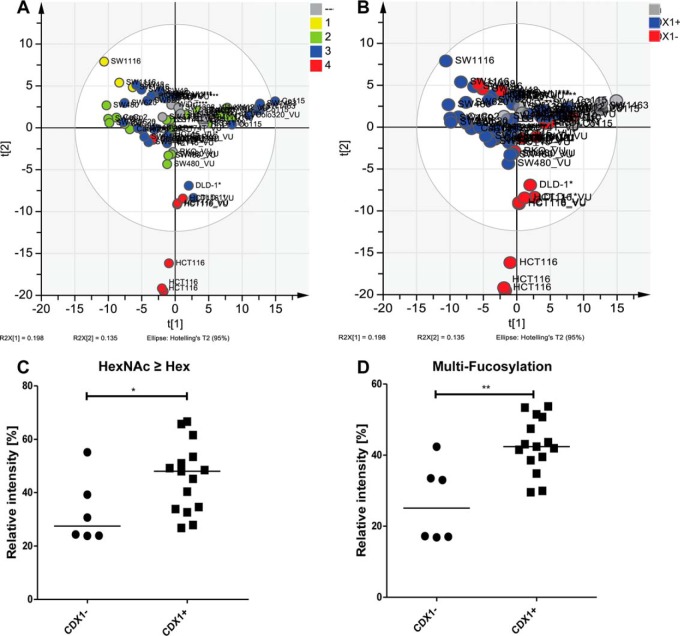
**Glycan trait correlations with CDX1/villin mRNA.** (*A*) PCA score plot of an unsupervised PCA model displaying principal components (PC) 1 and 2 (see Fig. 4*A*) colored according to stage of the original tumor. (*B*) Score plot of PC1 *versus* PC2 colored according to CDX1/villin-mRNA expression: CDX1/villin-positive (+) *versus* CDX1/villin-negative (-). Expression of mRNA of CDX1/villin was retrieved from (33–35). Potential glycan traits associated with CDX1/villin-mRNA expression based on loadings of the PCA model (See Figs 4*B* and 4*C*) and were calculated by summing relative intensities from MALDI-TOF-MS analysis for averages of 2–3 biological replicates. A Mann–Whitney nonparametric test with significance level α = 0.05 was performed. CDX1/villin-negative (CDX1-) and CDX1/villin-positive (CDX1+) were significantly different with increased levels in CDX1+ cells of C) *N*-glycans with higher amount of *N*-acetylhexosamine (HexNAc) than hexoses (Hex), *p* value .0471, and D) multi-fucosylation, *p* value .0026.

To gain more insight in the biology of the *N*-glycans, glycan traits were tested for association with cell line characteristics as stage of the original tumor and differentiation. Coloring the PCA scores of PC1 and PC2 corresponding to the stages of the tumors from which the cell lines originated showed a gradient from stage I toward stage IV (Fig. 5*A*), indicating differences in fucosylation as well as the ratio of Hex/HexNAc based on corresponding loading plots (Figs. 4*B* and 4*C*). However, statistical evaluation by a Kruskal–Wallis nonparametric test did not reveal significant differences in fucosylation nor Hex/HexNAc ratio between colorectal cancer stages (data not shown). Interestingly, each disease stage group contained two subgroups that were identified as CDX1/villin-positive and CDX1/villin-negative cell groups (data not shown). The homeobox gene CDX1 is an intestine-specific transcription factor associated with differentiation and villin expression and therefore was used as a marker for the differentiation state of the cell lines (35, 39). Coloring the PCA plot according to CDX1/villin-expression showed a separation within the first two principle components (Fig. 5*B*), and statistical analysis using Mann–Whitney test revealed CDX1/villin-expression to associate with a terminal HexNAc (Fig. 5*C*, *p* value .047) as well as multi-fucosylation (Fig. 5*D*, *p* value .003). Coexpression data from the Cancer Cell Line Encyclopedia supported this association partly and showed CDX1 positively correlated with FUT3 (Pearson's correlation 0.63; Supplemental Table S8). Furthermore, glycosyltransferase B3GNT8, involved in the generation of LacNAc repeats was positively correlated with CDX1 (Pearson's correlation 0.79; Supplemental Table S8). We observed a trend toward higher poly-antennarity, reflecting more than four LacNAc repeats, in CDX1/villin positive cells but no statistically significance (data not shown). However, the cell line SW48 appeared as an outlier not following this association and expressing a CDX1/villin+ glycan phenotype while being CDX1/villin-.

##### Glycosyltransferase Expression

In order to obtain information on enzyme expression, mRNA of 17 glycosyltransferases (GTs) were obtained by real-time PCR for a subset of cell lines (all cell lines obtained from the Leiden University Medical Center; Supplemental Table S7). The PCA resulted in five principle components explaining 84.7% (R^2^Xcum) of the data with a good prediction power of 56.1% (Q^2^cum). The cell lines clustered per cell type, while technical replicates clustered closely together. Notably, taking the GT gene expression data, the location of the cell lines differs from MS-based data and cell lines HCT116 and DLD-1 do not cluster distinctly apart from the other cell lines (Supplemental Fig. S7*A*). In contrast, C10, HCT8, WiDr, and SW1116 seem to be different from the other compared cell lines taking the measured GT into account. The corresponding loading plot (Supplemental Fig. S7*B*) suggests high branching of *N*-glycans (MGAT4B, MGAT5) as well as LacNAc repeats (B3GNT3,8) and α2,3-sialylation (ST3GAL3,4) for these latter cell lines, which is in accordance to obtained high gene expression levels (Supplemental Table S7). Cell lines SW1116 and WiDr, furthermore, have high levels of fucosylation in both the loading plot as well as mRNA data. CaCo2 localizes also apart from the majority of cell lines and shows in the loading plot only MGAT4A and partly ST3GAL6 associated (Supplemental Fig. S7*B*). The mRNA data support this observation since only MGAT4A and ST3GAL6 genes are highly expressed in CaCo2, while all other GT gene expressions are below the average of the tested cell lines (Supplemental Table S7).

Correlation analysis between derived glycan traits based on MS-data and corresponding GT mRNA levels showed significant correlation for mannosyl-(α1,3-)-glycoprotein-β-1,4-*N*-acetylglucosaminyltransferase, isozyme A (MGAT4A; R^2^ = 0.363, *p* value = .008), involved in the synthesis of tri- and multi-antennary glycans and the relative abundance of the corresponding glycan class (Supplemental Fig. S7*C*), as well as for fucosyltransferase (FUT) 4, the enzyme responsible for Lewis X epitopes on LacNAc repeats, with the trait multi-fucosylation (R^2^ = 0.2947, *p* value = .020; Supplemental Fig. S5*D*). Furthermore, β-galactoside-α2,3-sialyltransferase 4 (ST3GAL4), involved in the production of sialyl Lewis X, showed significant correlation (R^2^ = 0.236, *p* value = .048) with the relative abundance of α2,3-linked sialic acid containing *N*-glycans based on MS data (Supplemental Fig. S7*E*). Overall, the mRNA data showed more overlap for GTs specifically involved in the *N*-glycan biosynthesis rather than with enzymes contributing to the biosynthesis (elongation/decoration) of more glycan classes, *i.e. N*-glycans, *O*-linked glycans and/or glycosphingolipids. However, FUT8, the enzyme responsible for α1,6-fucosylation of the *N*-glycan core, showed no correlation with the derived trait mono-fucosylation and instead showed the highest levels for SW48, SW480, SW620, and WiDr. This may indicate that, although MS/MS data suggested mainly core-fucosylated structures, mono-fucosylation is not only representative for core-fucosylation but also antenna-fucosylation. The discrepancy between GT gene expression and MS data shows that the value of GT expression data for predicting actual glycan structures is rather limited and that a better understanding of factors influencing protein glycosylation may be needed to improve this prediction.

## DISCUSSION

We investigated the *N*-glycome of 25 CRC cell lines including 21 cell lines derived from primary tumors and four from metastases revealing vast glycosylation differences between the cell lines. By applying a PVDF-membrane-based *N*-glycan release in combination with a new derivatization technique for linkage-specific stabilization of sialic acids, we achieved a sensitive high-throughput MALDI-TOF-MS analysis of *N*-glycans from small numbers of cells. The method proved to be robust for data acquisition while the calculated, derived glycan traits showed an even more pronounced robustness than most individual glycans. Moreover, the differentiation between α2,3- and α2,6-linked *N*-acetylneuraminic acids by linkage-specific derivatization adds major biological relevance to the results as it allows detection of the presence of sialyl Lewis antigens that are highly associated with cancer (40).

Often, physiological conditions are disrupted when cells are cultured *in vitro* without being in their natural surrounding containing stromal and other cells. The main critical points of cell lines as model systems are therefore the following: (i) Culturing conditions may influence the cellular phenotype. (ii) Tumor heterogeneity is not represented. (iii) The influence of the tumor environment is not given (15). Nevertheless, it was shown that genetic profiles as well as functional markers and morphological features are commonly retained for CRC cell lines (12, 41). Also, our study revealed a major overlap in the expression of complex-type *N*-glycans between CRC cell lines and CRC tissue samples (10, 11) as well as the expression of CRC/colon-associated epitopes such as (sialyl) Lewis antigens, the Sda antigen, blood group antigens, and terminal HexNAc, thereby revealing the potential of these CRC cell lines as *N*-glycomic model system. Similarly, high correlations between tissues and cell lines were described on the genomic level (17, 19, 41). Notably, the *N*-glycan profiles of the majority of analyzed CRC cell lines were dominated by high-mannose-type glycans (Ø 53.0% in our study), which is in accordance with recently reported data by the group of N. Packer (Ø 55.0% high-mannose *N*-glycans) (22) but which represents a major difference to *N*-glycans derived from CRC tissues. However, it should be noted that in comparison to control tissues, CRC tissues also exhibit elevated levels of high-mannose-type glycans (10). Similar trends were also observed in breast cancer (42), and also human stem cells show a high abundance of high-mannose-type *N*-glycans (43). From a biosynthesis point of view, the accumulation of high-mannose-type glycans in the CRC cell lines and tissues suggests an incomplete processing of *N*-glycans, possibly due to shorter division/replication times (44). Especially the glycan Man9HexNAc2Glc1 (*m/z* 2067.69) gives evidence to the presence of intracellular precursors in the analyzed total cell homogenate glycan pools. Nevertheless, lectin-binding data showed strong interaction of several CRC cell lines with ConA (data not shown) suggesting the presence of high-mannose-type glycans. Of note, this lectin is not entirely specific for high-mannose *N*-glycans and can also bind to hybrid and di-antennary *N*-glycans, albeit with lower affinities (45). Investigations on the cell surface glycosylation of a subset of cell lines showed as well spectra with dominant peaks of high-mannose-type glycans, proving the presence of high-mannose-type glycans on the surface but to a lower relative abundance as compared with their accumulation in whole cell homogenates as recently also shown for HEK cells (26). This cell surface protocol could not be performed in 96-well plates and involved large amounts of highly active PNGase F to allow short incubation times of 30 min in order to shave the glycans from the cell surface. This makes it less high-throughput suited and very costly. Furthermore, 4 million cells are needed for this protocol, which for some cell types is not achievable. Therefore, although containing intracellular glycans, our method offers a good and reliable alternative for the screening of *N*-glycosylation of cell lines and is suited for small sample amounts. To address the presence of intracellular precursors, high-mannose-type glycans were excluded for a major part of the trait calculations in order to observe true changes in complex type *N*-glycans.

In this study, we identified the presence of Sda antigen (NeuAcα2–3[GalNAcβ1–4]Galβ1–4GlcNAc-R) in Caco2 cell lines based on MS data, while mRNA data on the corresponding GT, B4GALNACT2 showed elevated levels for WiDr and SW1116 cells and not for CaCo2. The latter result is in contrast to previous findings by Dall'Olio and coworkers who detected B4GALNACT2 mRNA exclusively expressed in CaCo2 when tested with eight other CRC cell lines (not WiDr and SW1116) (46). They describe a varying activity of this GT and suggest B4GALNACT2 as marker of the colonic cell maturation (46). Furthermore, the group around Dall'Olio showed the inhibition of sialyl Lewis X formation through Sda antigen expression with LS174T colon cancer cells as model system (47). To our knowledge, there is no literature on the presence of Sda antigen in WiDr and SW1116.

The studies of Packer and coworkers on the *N*-glycome of membrane proteins from various CRC cell lines as well as CRC tissues using porous graphitized carbon LC-ESI-MS/MS (22, 48) showed that the metastatic CRC cell line LIM1215 presents high levels of *N*-glycans containing bisecting GlcNAc as compared with two nonmetastatic cancer cell lines. We, as well, observed high levels of potentially bisected complex or hybrid type *N*-glycan structures (Hex = HexNAc) in SW620 (38.4%), a cell line derived from lymph node metastasis. This is in contrast to literature, often describing the role of bisecting GlcNAc in suppressing metastasis (49). Moreover, the expression of MGAT3, the enzyme initiating bisection, was very low in SW620, suggesting that the suspected bisection is more likely to reflect terminal HexNAc on antennae. Also, our data did not show enhanced levels of potential bisection (Hex = HexNAc) exclusively for cell lines of metastatic origin but also in the well-differentiated stage I cell line SW1116 (51.2%) for which the presence of bisecting GlcNAc was confirmed by LC-MS/MS experiments and also MGAT3 gene expression was largely elevated. This is in accordance with another study of Packer and coworkers describing high expression (15%) of bisecting GlcNAc for the cell lines SW1116, but also SW620, and only very low levels for SW480 (22). In line, our data showed very low expression levels of MGAT3 for SW480 and no indication for bisection was found in MS/MS. However, the discrepancy between enzyme gene expression and relative abundance of bisection in some of the cell lines reveal the limit of the applied MS-based methods as it cannot in all cases sufficiently be differentiated if the additional HexNAc represents a bisecting GlcNAc or a terminal HexNAc. An additional method may be offered by a novel lectin that recognizes terminal nonreducing GlcNAc residues, also on galactoses of *N*-glycan antennae, and was shown to stain colon cancer tissues and cell lines (HT29), whereas no or weak staining was observed in healthy tissues (60).

Regarding sialylation, we observed a higher level of α2,3-sialylation and a decreased percentage of α2,6-sialylation in CDX1-positive cells that correlate with cell differentiation. In contrast, Sethi and collaborators reported on the presence of α2,3-sialylation exclusively in the poorly differentiated cell line LIM2405 (48). In general, overall levels of observed sialylation and fucosylation differ between this study and the studies of Packer and coworkers (22, 48). Such dissimilarities in the results are not surprising since different methods of glycan extraction and purification as well as different analytical techniques—MALDI-TOF-MS(/MS) *versus* porous graphitized carbon LC-ESI-MS(/MS)—were applied. In addition, different culture conditions may play a role since it has been shown that the protein glycosylation is influenced by cellular, medium, and process effects, which is frequently addressed for the production of glycoproteins in the pharmaceutical industry (51). Also in our study, we found differences in the *N*-glycosylation profile between cell lines cultured in different laboratories and the impact of various factors on the glycan profile during the cell culture needs to be further investigated—not only for pharmaceutical productions but also for *in vitro* experiments with cancer cell lines in order to prevent misleading interpretation of results.

Interestingly, we observed a correlation between multi-fucosylation and expression of CDX1 and/or villin. CDX1 is an intestine-specific transcription factor associated with differentiation and villin expression (34, 39). Investigations on cancer stem cell subgroups in CRC cell lines revealed a higher portion of cancer stem cells in nondifferentiating cell lines such as HCT116, which is accompanied with loss of CDX1 expression and a more aggressive phenotype (50). After forcing CDX1 expression in HCT116 cells, intestinal epithelial differentiation to crypt-forming colonies was observed (50). Furthermore, cell line HCT116 has a mutation in the GDP-mannose-4,6-dehydratase (GMDS) and therefore very low fucose-levels and showed an aggressive phenotype, while restoration of the GMDS transcript and therefore enhanced fucosylation suppressed tumor formation and metastasis (52). Enhanced fucosylation was further described in early stages of cancer, while lower fucosylation was associated with later stages and cancer progression (53). In accordance, complex fucosylation decreased from colorectal adenomas toward carcinomas (11). In contrast, high antenna fucosylation of TGF-β was associated with poor prognosis and metastasis (54). We further found a correlation between CDX1/villin expression and glycans with terminal HexNAc (HexNAc≥Hex ratio), in form of terminal HexNAc, bisecting GlcNAc, or LacdiNAc structures, and a trend (not significant, data not shown) toward higher antennarity/LacNAc repeats was observed for CDX1/villin-positive cells. In line, Kawasaki *et al.* (55) identified highly fucosylated polylactosamine-type *N*-glycans in a CRC cell line SW1116 that are expressed specifically on CD26/dipeptidyl peptidase IV and serve as ligands for mannan-binding protein, a C-type lectin involved in host defense and tumor growth inhibition in CRC cells. In this study, we found that SW1116, a well-differentiated cell line derived from a primary stage I tumor, is characterized by an overall enhanced presence of polylactosamine repeats as well as high levels of multi-fucosylation. Supporting the finding of Kawasaki *et al.*, Chen *et al.* (56) reported that expression of β-1,4-galactosyltransferase 3, involved in the synthesis of (poly)lactosamine epitopes, significantly suppresses β1-integrin-mediated cell migration and invasion. For this subset of CRC cell lines, our findings in combination with other reports in literature support the idea that multi-fucosylation, and possibly also (poly)lactosamine repeats, are characteristics of differentiated cell lines expressing CDX1 and villin and exhibiting a less invasive, less aggressive phenotype. Notably, SW48 cells behave as outliers and show a more CDX1/villin-positive *N*-glycan phenotype. Clearly, further glycomic studies on more CRC cell lines as well as CDX1-knockdown cell lines and cells with forced CDX1 expression are needed to evaluate whether CDX1 acts as an inducer of the associated glycomic profiles, and assays on aggressiveness/invasiveness of corresponding cell lines are required to show the biological relevance.

The analysis of the CRC cell lines with regard to their *N*-glycome provided new insight in their glycosylation features that are lacking behind genetics and helps to evaluate cell lines as glycobiological model system. However, in order to choose an optimal model system, a larger-scale study comparing cell line glycosylation with tissue-derived glycan profiles is needed. Also, we observed major differences between the cell lines with regard to their *N*-glycosylation, demonstrating that the study of a single CRC cell line model can lead to the wrong generalization of glycobiological findings. Moreover, the similarity with regard to other glycan classes needs to be further studied, and the first results on *O*-glycosylation (22) showed major differences between CRC cell lines and tumor tissues. Therefore, cell lines need to be well characterized in all aspects, and assumptions and interpretations should refer only to the cell phenotype that has been studied. The high heterogeneity of cell line glycosylation can complicate the interpretation and comparison of results but also offers a major advantage. One of the main criticisms on cell lines as model systems is that they lack the ability to represent tumor heterogeneity. Since tumor heterogeneity can hardly be displayed in one single cell line, combining different cell lines *in vitro* might aid to mimic the tumor. Collecting detailed glycomic data on tumor subpopulations in a database can facilitate this approach by matching the characteristics to available cell line glycomic data and therefore improve the potential of cell lines as model systems.

## Footnotes

^1^ The abbreviations used are:

CRC: colorectal cancer
DHB: 2,5-dihydroxybenzoic acid
dHex: deoxyhexose
DMEM: Dulbecco's Modified Eagle
F: Fuc, fucose
FCS: fetal calf serum
GalNAc: *N*-acetylgalactosamine
GlcNAc: *N*-acetylglucosamine
GuHCl: guanidine hydrochloride
H: Hex, hexose
N: HexNAc, *N*-acetylhexosamine
HILIC: hydrophilic interaction liquid chromatography
LC-ESI-MS: liquid chromatography-electrospray ionization-mass spectrometry
MALDI-TOF-MS: matrix-assisted laser desorption/ionization time-of-flight mass spectrometry
MS/MS: tandem mass spectrometry
NeuAc: *N*-acetylneuraminic acid
PCA: principal component analysis
PNGase F: peptide *N*-glycosidase F
RPMI: Roswell Park Memorial Institute
SPE: solid phase extraction.
